# A Neurotoxic Phosphoform of Elk-1 Associates with Inclusions from Multiple Neurodegenerative Diseases

**DOI:** 10.1371/journal.pone.0009002

**Published:** 2010-02-02

**Authors:** Anup Sharma, Linda M. Callahan, Jai-Yoon Sul, Tae Kyung Kim, Lindy Barrett, Minsun Kim, James M. Powers, Howard Federoff, James Eberwine

**Affiliations:** 1 Department of Neuroscience, University of Pennsylvania, Philadelphia, Pennsylvania, United States of America; 2 Department of Pathology and Laboratory Medicine, University of Rochester Medical Center, Rochester, New York, United States of America; 3 Department of Pharmacology, University of Pennsylvania, Philadelphia, Pennsylvania, United States of America; 4 Department of Cancer Biology and Genetics, Memorial Sloan-Kettering Cancer Center, New York, New York, United States of America; 5 Department of Physiology, Wonkwang University School of Medicine, Iksan, South Korea; 6 Department of Neurology, Georgetown University Medical Center, Washington, D.C., United States of America; 7 Penn Genome Frontiers Institute, University of Pennsylvania, Philadelphia, Pennsylvania, United States of America; Tokyo Medical and Dental University, Japan

## Abstract

Neurodegenerative diseases are characterized by a number of features including the formation of inclusions, early synaptic degeneration and the selective loss of neurons. Molecules serving as links between these shared features have yet to be identified. Identifying candidates within the diseased microenvironment will open up novel avenues for therapeutic intervention. The transcription factor Elk-1 resides within multiple brain areas both in nuclear and extranuclear neuronal compartments. Interestingly, its *de novo* expression within a single dendrite initiates neuronal death. Given this novel regionalized function, we assessed whether extranuclear Elk-1 and/or phospho-Elk-1 (pElk-1) protein might be associated with a spectrum of human neurodegenerative disease cases including Lewy body Disease (e.g. Parkinson's), Alzheimer's disease, and Huntington's Disease. We first determined the importance of Elk-1 post-translational modifications on its ability to initiate regionalized cell death. We next screened human cases from three major neurodegenerative diseases to look for remarkable levels of Elk-1 and/or pElk-1 protein as well as their association with inclusions characteristic of these diseases. We compared our findings to age-matched control cases. We find that the ability of Elk-1 to initiate regionalized neuronal death depends on a specific phosphosite, T417. Furthermore, we find that T417^+^ Elk-1 uniquely associates with several types of inclusions present in cases of human Lewy body Disease, Alzheimer's disease, and Huntington's Disease. These results suggest a molecular link between the presence of inclusions and neuronal loss that is shared across a spectrum of neurodegenerative disease.

## Introduction

Elk-1 is a member of the ternary complex factor (TCF) subfamily of ETS-domain transcription factors [1]. It is targeted in the nucleus by three major MAP kinase pathways as well as the CaMKII pathway [2]. Phosphorylation of nuclear Elk-1 enhances its ability to bind DNA at serum response elements and stimulate transcription of immediate early genes [3]. Within this functional context, nuclear Elk-1 has been implicated in processes such as neuronal differentiation [4], cellular proliferation [5] and tumorigenesis [6].

There is growing evidence that Elk-1 has broader functional properties. Expression analysis reveals robust Elk-1 expression within diverse brain regions including the cortex, hippocampus, striatum and cerebellum [7]. Subcellular localization analysis shows Elk-1 mRNA and protein to be present in both nuclear and extranuclear compartments including the neuronal soma and dendrites [8]. Furthermore, post-translationally modified forms of Elk-1 are also present in extranuclear neuronal compartments [8]. This diversity in Elk-1 cellular and subcellular localization implies diversity in Elk-1 function.

We previously reported the association between Elk-1 and the mitochondrial permeability transition pore complex (PTP) [9]. The PTP is a protein complex that forms a functional pore across mitochondrial membranes. Activation of this pore is believed to induce both apoptosis and necrosis[10]. In our previous report, co-IP experiments show that Elk-1 associates with multiple components of the PTP. Electron microscopy of adult rat brain sections show that this association is present in both neuronal soma and dendrites. Furthermore, the introduction of pro-apoptotic stimuli enhances the association between Elk-1 and mitochondria in primary neurons[9].

We previously reported that the *de novo* synthesis of Elk-1 protein within a single neuronal dendrite initiates cell death[8]. Here, we used phototransfection to focally introduce and express small amounts of mRNA into various compartments of a single primary hippocampal neuron. Following phototransfection and expression of Elk-1 mRNA within dendrites, the majority of neurons undergo cell death that can be seen as early as thirty minutes to several hours later. Phototransfection with Elk-1 GFP mRNA shows that Elk-1 translocates from the dendrite into the nucleus as part of the cell death process. Pharmacological inhibition of transcription prevents Elk-1 mediated neuronal death. In addition, pharmacological inhibition of the PTP prevents Elk-1 mediated neuronal death. These findings describe a unique role for extranuclear Elk-1 protein in regulating neuronal viability through interactions with the nucleus and mitochondria.

**Figure 1:**
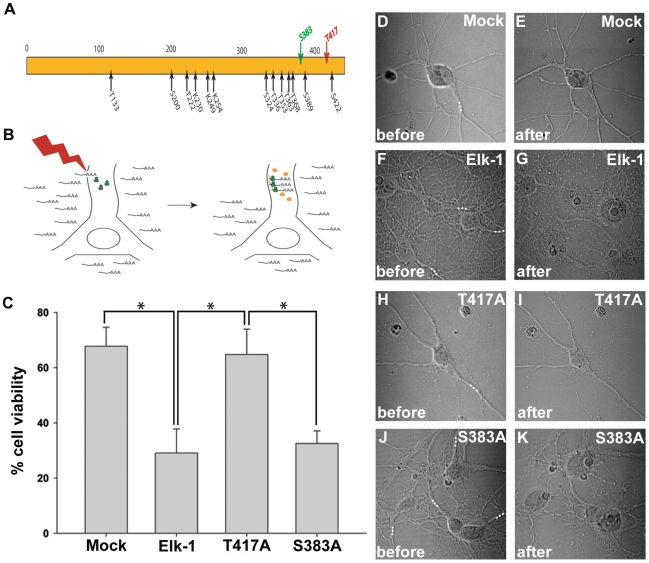
Elk-1 mediated neuronal death is prevented by a specific phosphosite mutation. Elk-1 protein contains fifteen sites of post-translational modification (Panel A). Phototransfection introduces small amounts of mRNA into various compartments of a single primary hippocampal neuron allowing for localized protein expression (Panel B). Primary hippocampal neurons were phototransfected within a neuronal dendrite under mock conditions (Panel D, n = 90), with Elk-1 mRNA (Panel F, n = 105), T417A Elk-1 mRNA (Panel H, n = 80) and S383A Elk-1 mRNA (Panel J, n = 49). Cell death was determined based on cellular morphology (Panel E, G, I, K). White dots represent the regions targeted for phototransfection. A graph compares % cell viability following phototransfection with the different mRNAs (Panel C). (error bars  =  SEM, *P<0.05).

Given that extranuclear Elk-1 has novel functional properties, we wanted to investigate whether post-translational modifications could be contributory. Elk-1 contains fifteen sites of post-translational modification (Figure 1A). The majority of these sites can be modified *in vitro* or *in vivo* [11], [12], [13], [14], [15]. However, only a few sites have been implicated in regulating Elk-1 function and much of this work has been performed within non-neuronal systems. Thus, the functional significance of most of these sites is unknown especially within neuronal systems. For these reasons, we wanted to determine whether one or more of these sites could be important in regulating extranuclear Elk-1 function. Specifically, we tested whether any of these sites were essential for Elk-1 to mediate neuronal death.

Despite having unique clinical and pathological characteristics, neurodegenerative diseases are characterized by the abnormal aggregation of proteins and the formation of inclusions[16], [17]. In addition, the earliest signs of neuronal degeneration are thought to occur locally in the synapses and the neurites [18]. Furthermore, they are associated with the selective loss of neurons, though the mechanism(s) are unknown [16], [17]. Given that Elk-1 expresses within multiple brain areas [7] and its *de novo* dendritic protein synthesis leads to neuronal death[8], we wanted to determine whether Elk-1 and/or one of its phosphoforms (pElk-1) could be involved in neuronal death associated with neurodegenerative disease. We tested for remarkable Elk-1 and/or pElk-1 protein in the classic brain regions undergoing clinically significant neuronal loss in either human Lewy Body disease (e.g. Parkinson's Disease), Alzheimer's disease (AD), or Huntington's Disease (HD). Specifically, we tested for an association between Elk-1 and/or pElk-1 protein with inclusions present in these diseases, the areas that indicate disease pathogenesis.

## Results

### Elk-1 Neuronal Death Is Phosphosite-Dependent

We first assessed whether Elk-1 mediated neuronal death depends on one or more sites of post-translational modification. Since phototransfection introduces only a small amount of Elk-1 mRNA into a single dendrite of primary neurons, the phosphorylation status of *de novo* synthesized Elk-1 cannot be detected. Consequently, mutational and functional analysis was utilized to determine the importance of these sites on *de novo* synthesized Elk-1. Here, site-directed mutagenesis created Elk-1 mRNA mutated at a single site. Then, each mutant mRNA was separately phototransfected into neuronal dendrites to determine its effects on cell viability in comparison to wildtype Elk-1 mRNA and a mock phototransfection without mRNA (Figure 1B). Our preliminary screen showed that the majority of mutant mRNAs were able to recapitulate wildtype Elk-1 mediated cell death suggesting that their respective sites were not essential in the cell death process. However, select mutant mRNAs were not effective in initiating neuronal death. We selected one mutant mRNA from each group for a more thorough analysis including S383A Elk-1 (caused cell death to a similar extent as native Elk-1) and T417A Elk-1 (the mutant, that in the initial screening, most consistently did not cause cell death). Primary hippocampal neurons were phototransfected within single dendrites with either wildtype Elk-1 mRNA or one of the two mutant mRNAs. Cell viability was determined based on morphology within six hours of phototransfection (Figure 1C). Following phototransfection of wildtype Elk-1 mRNA, the majority of neurons undergo cell death within hours that is characterized by dendritic fragmentation and somatic swelling (Figures 1F–G). Phototransfection of S383A Elk-1 mRNA initiates cell death with similar temporal and morphological characteristics in a majority of neurons (Figure 1J–K). Differences in percent cell viability following phototransfection of S383A Elk-1 mRNA compared to phototransfection with wildtype Elk-1 mRNA were not statistically significant (Figure 1C). In contrast, following phototransfection of T417A Elk-1 mRNA, the majority of neurons do not show a cell death phenotype within six hours (Figure 1H–I), similar to mock phototransfection (Figure 1C). These experiments demonstrate that Elk-1 mediated neuronal death is prevented by a specific phosphosite mutation at T417. They suggest that T417^+^ Elk-1 protein is essential in mediating neuronal death.

We next assessed whether there could be an association between Elk-1 and/or pElk-1 protein with inclusions present in neurodegenerative disease. Here, cases were selected based upon their final postmortem diagnosis, which was either a variant of Lewy Body Disease (e.g. Parkinson's Disease), Alzheimer's Disease or Huntington's disease. Postmortem brain tissue from age-matched control patients was also included for comparison. Table S1 includes relevant disease characteristics about the patients selected for this study.

### Elk-1 and Lewy Body Disease

**Figure 2:**
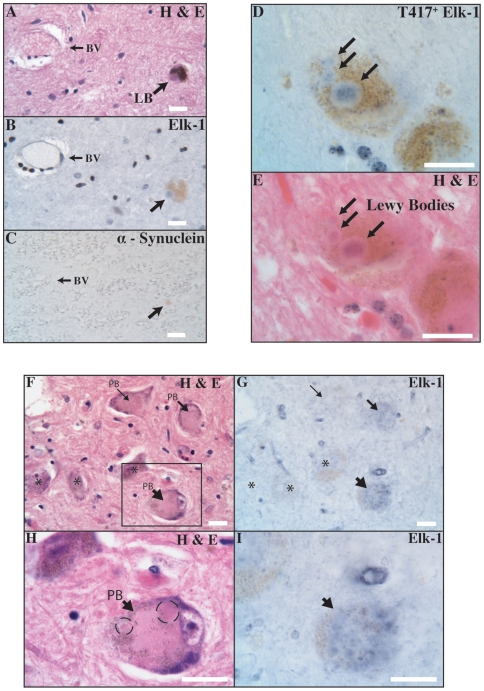
T417^+^ Elk-1 co-localizes with Lewy Bodies and pale bodies in human Lewy Body Disease tissue. A classic Lewy body in a neuromelanin-containing substantia nigra neuron (arrow in Panel A) also stains with Elk-1 (bluish-black, arrow in Panel B) and alpha-synuclein (red, arrow in Panel C) in immediately adjacent sections (patient LB7; scale bars, 20 µm; original magnification, 400×). A SN neuron contains multiple T417^+^ Elk-1 inclusions (bluish-black, arrows in Panel D). Following an acid removal procedure the section is overlaid with H&E. T417^+^ Elk-1 inclusions exhibit dark pink eosinophilic centers surrounded by lighter pink halos (patient LB6; scale bars, 20 µm; original magnification, 1000×). A SN section contains neurons with pale bodies (arrows in Panel F) and neurons that do not (asterisks in Panel F). The corresponding Elk-1immunoreactivity is shown for these neurons (Panel G) (patient LB4, scale bars, 20 µm; original magnification, 400×). A higher magnification of a pale-body containing neuron (arrow in Panel H) with its corresponding Elk-1immunoreactivity (arrow in Panel I) (scale bars, 20 µm; original magnification, 1000×).

We first tested whether Elk-1 protein can co-localize with Lewy bodies in brain tissue from patients with Lewy Body Disease. A cohort of cases representing a spectrum of Lewy Body Disease with a wide range in pathology was selected for this analysis. The cases exhibit a wide range in the number of neurons containing Lewy bodies as well as the severity of neuronal loss (Table S2). The cases examined here include patients with subcortical predominant Lewy bodies (i.e. classical Parkinson's Disease), patients with neocortical involvement (i.e. Diffuse Lewy Body Disease, Lewy Body Disease with Dementia) as well as patients with a preclinical presentation of Lewy Bodies (Methods S1). Figure 2A–C contains representative images demonstrating co-localization of Elk-1 with a Lewy body in tissue from a patient with Lewy Body Disease. Using a stringent staining paradigm, serially adjacent 7 µm sections containing substantia nigra were processed with H&E (Figure 2A), an antibody against total Elk-1 (Figures 2B), and an antibody against alpha-synuclein (Figure 2C). Arrows demonstrate that a classic Lewy body in a neuromelanin-containing neuron (arrow in Figure 2A) also contains Elk-1 (arrow in Figure 2B) and alpha-synuclein (arrow in Figure 2C). Elk-1 immunoreactivity, assessed in the middle adjacent section, localizes to the central core region of the Lewy body and not the halo. A nearby blood vessel is used for cross-sectional XY alignment. Elk-1 signal is present in each of the Lewy bodies imaged in Figure 2. This is apparent by comparing the signals from Figure 2 with those Lewy bodies lacking signal in Figure S1. Overall, a subset of Lewy bodies show Elk-1 co-localization. Here, the level of staining and the degree of Lewy body co-localization can depend on a number of detection and disease variables [19], [20]. Despite using a conservative staining paradigm, multiple examples of co-localization were identified, including from each Lewy Body Disease variant listed in Table S1.

We next tested whether any phosphoforms of Elk-1 were present in these inclusions. Here, we used an antibody against S383^+^ Elk-1, a phosphoform studied within the context of nuclear Elk-1 function. We also used an antibody against T417^+^ Elk-1, a phosphoform identified as likely being essential for regionalized neuronal death. Finally, we used an antibody against T368^+^ Elk-1, a phosphoform of unknown functional relevance.

Figure 2D–E includes representative images demonstrating that T417^+^ Elk-1 inclusions display classic Lewy body morphologies in tissue from a patient with Lewy Body Disease (LBD). T417^+^ Elk-1 immunoreactivity stains the core region of multiple intracellular inclusions in a neuromelanin-containing neuron (arrows in Figure 2D). When overlaid with H&E, the inclusions exhibit classical dark pink eosinophilic centers surrounded by lighter pink halos characteristic of Lewy bodies (arrows in Figure 2E). The co-localization of T417^+^ Elk-1 with Lewy bodies is present in tissue from other variants of Lewy Body Disease. However, similar experiments utilizing antibodies specific for S383^+^ Elk-1 and T368^+^ Elk-1 do not show co-localization with Lewy bodies (Figure S1 A/B). Substantia nigra tissue from age-matched control patients does not contain T417^+^ Elk-1 inclusions. Occasional neurons do exhibit significant immunostaining for T417^+^ Elk-1 (Figure S2 A/B, arrow). Interestingly, strong nuclear T417^+^ Elk-1 immunoreactivity is identified within substantia nigral glial cells (Figure 2B).

Pale bodies are thought to be precursors of Lewy bodies and have been shown to share a subset of antigenic determinants[21]. Figure 2F-I shows that within a group of substantia nigra neurons from a patient with a preclinical presentation of LBD, a subset of neurons contains pale bodies (Figure 2F; arrows). These intracytoplasmic inclusions are characterized by their larger size, light glassy eosinophilic character and absence of halos. Of the three neurons containing pale bodies, two show increased Elk-1 expression in the pale body regions (Figure 2G; thicker arrows) compared to the neurons lacking pale bodies (Figure 2G; asterisks). Interestingly, the neuron with the pale body displaying the most intense immunostaining for Elk-1 (Figure 2I) appears to be at a later stage of inclusion development, in that it contains multiple early Lewy bodies at its periphery (dashed ellipses, Figure 2H). In addition to total Elk-1, T417^+^ Elk-1 is also present in pale bodies. In sum, these experiments show that T417^+^ Elk-1 specifically associates with inclusions characteristic of human Lewy body disease tissue.

### Elk-1 and Alzheimer's Disease

**Figure 3:**
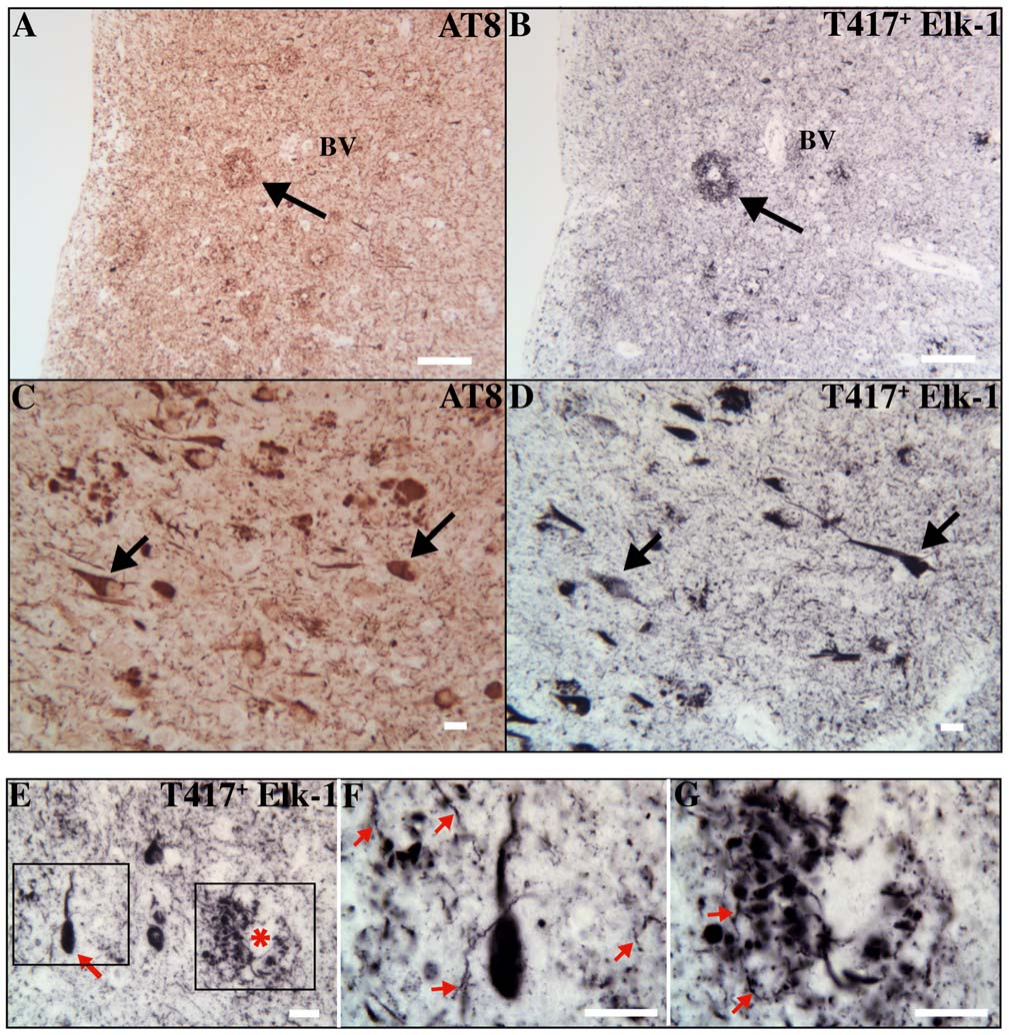
T417^+^ Elk-1 co-localizes with plaque and tangle inclusions in human Alzheimer's Disease tissue. A parahippocampal neuritic plaque identified by AT8 (arrow in Panel A) also contains T417^+^ Elk-1 immunoreactivity (arrow in Panel B) (patient AD2; scale bars, 200 µm; original magnification, 40×). Neurofibrillary tangles identified by AT8 (arrows in Panel C) also contain T417^+^ Elk-1 immunoreactivity (arrows in Panel D) (scale bars, 20 µm; original magnification, 200×). T417^+^ Elk-1 inclusions show neurofibrillary tangle morphology (arrow in Panel E), neuritic plaque morphology (asterisk in Panel E), as well as dystrophic neurite morphology (neuropil in Panel E). (patient AD2; scale bar, 20 µm; original magnification, 400×). A higher magnification of a T417^+^ Elk-1 neurofibrillary tangle surrounded by multiple T417^+^ Elk-1 dystrophic neurites (arrows in Panel F) (scale bar, 20 µm; original magnification 1000×). A higher magnification of a T417^+^ Elk-1 plaque containing multiple T417^+^ Elk-1 dystrophic neurites (arrows in Panel G). (scale bar, 20 µm; original magnification, 1000×).

We tested whether a specific association exists between T417^+^ Elk-1 and inclusions in another major neurodegenerative disease. Alzheimer's Disease is characterized by a number of neuropathological lesions, including neuritic plaques, neurofibrillary tangles, and neuropil threads. These lesions can be identified by AT8, an antibody directed against hyperphosphorylated Tau. Figure 3A–D contains representative images showing that T417^+^ Elk-1 co-localizes with inclusions identified by AT8 in human Alzheimer's disease brain. A neuritic plaque labeled with AT8 immunostains intensely for T417^+^ Elk-1 in an adjacent section (arrow, Figure 3A/3B). In addition, a subset of neurons containing neurofibrillary tangles labeled with AT8 (arrows, Figure 3C), also immunostains intensely for T417^+^ Elk-1 in an adjacent section (arrows, Figure 3D). T417^+^ Elk-1 immunoreactivity also identifies numerous neuropil threads throughout the section (Figure 3D). This is consistent with the pattern of AT8 staining in the neuropil of Alzheimer's disease brain (Figure 3C). Furthermore, Figure 3E–G contains representative images demonstrating that T417^+^ Elk-1 inclusions display classic plaque, tangle and thread morphologies in human AD brain. The co-localization of T417^+^ Elk-1 with AD inclusions is found in each AD case listed in Table S1.

Immunostaining with antibodies against S383^+^ Elk-1 and T368^+^ Elk-1 do not demonstrate co-localization with AT8-labeled inclusions (Figure S3 A–C). Age-matched control tissue shows little inclusion pathology. Occasional neurons do exhibit significant extranuclear T417^+^ Elk-1 immunostaining (Figure S2 C/D, Figure S2 E/F; thick arrows). In sum, these experiments show that T417+ Elk-1 specifically associates with neuronal inclusions characteristic of Alzheimer's Disease.

**Figure 4:**
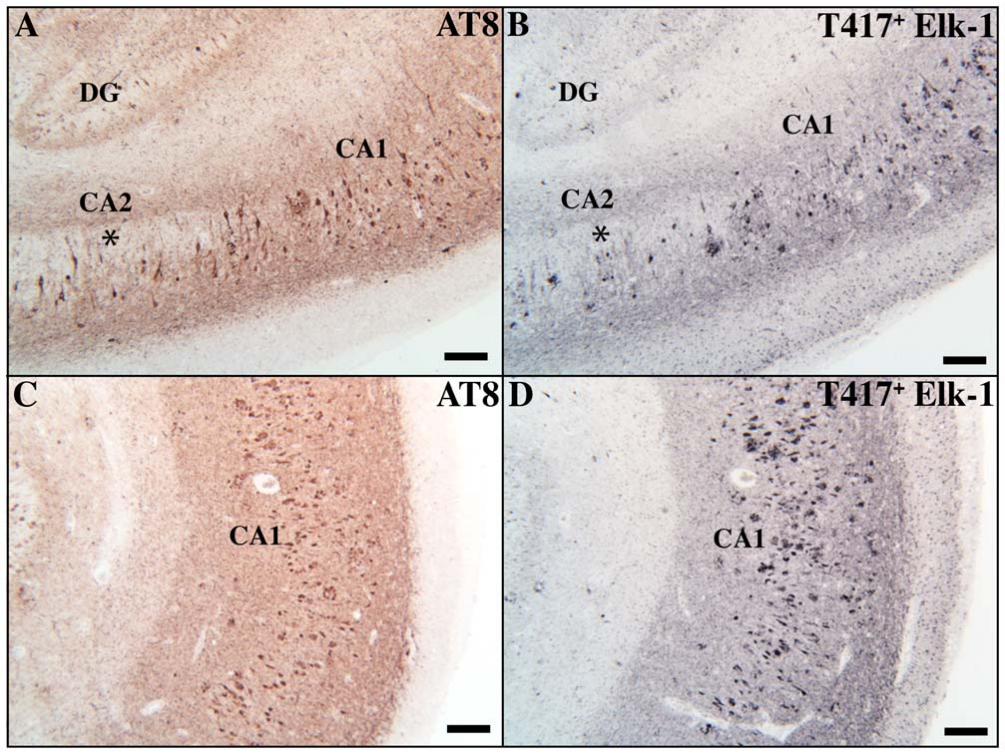
T417^+^ Elk-1 immunoreactivity mimics hippocampal regional pathology in human AD tissue. Serially adjacent sections containing AD hippocampus demonstrating the expression pattern of AT8 (Panels A,C) and T417^+^ Elk-1 (Panels B,D). Note the sparse tangle population in CA2 is identified by both AT8 (asterisk in Panel A) and T417^+^ Elk-1 (asterisk in Panel B) (patient AD2; scale bars, 200 µm; original magnification 40×). Abundant inclusion pathology in CA1 is identified by both AT8 (Panel C) and T417^+^ Elk-1 (Panel D). (scale bars, 200 µm; original magnification, 40×).

In Alzheimer's Disease, different areas of the hippocampus display distinct regional pathologies in terms of the number and distribution of inclusions [22], [23]. We find that T417^+^ Elk-1 immunoreactivity specifically mimics this regional pathology (Figure 4). In the CA2 region of hippocampus, note the sparse tangle population zone identified by both AT8 (Figure 4A, asterisk) and T417^+^ Elk-1 (Figure 4B, asterisk). In addition, note the high density of plaque and tangle staining in CA1 identified by both AT8 and T417^+^ Elk-1 staining (Figure 4C/4D). Robust neuritic inclusion staining is observed throughout the section. Finally, hippocampus from age-matched control tissue processed for T417^+^ Elk-1 does not show advanced CA1 pathology or a sparse tangle zone in CA2 (Figure S2C).

### Elk-1 and Huntington's Disease

**Figure 5:**
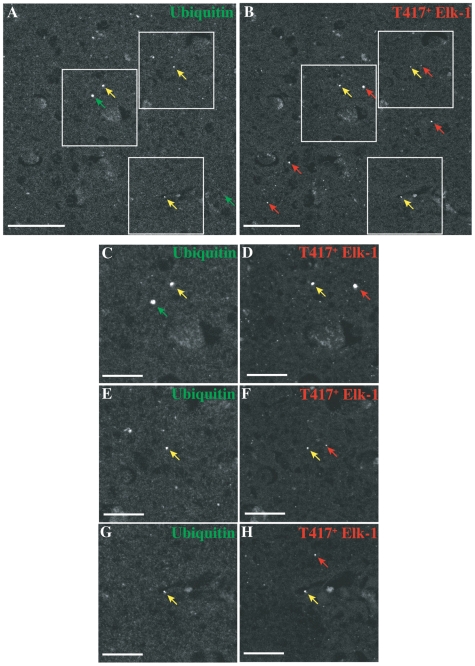
T417^+^ Elk-1 co-localizes with Ubiquitin^+^ inclusions in human HD tissue. Striatal inclusions identified by Ubiquitin^+^ immunoreactivity (yellow arrows in Panel A) also contain T417^+^ Elk-1 immunoreactivity (yellow arrows in Panel B) (patient HD7; scale bars, 50 µm). Green arrows identify inclusions only containing ubiquitin. Red arrows identify inclusions only containing T417^+^ Elk-1. Higher magnification images of insets (Panels C–H) (scale bars, 20 µm).

Finally, we assessed whether T417^+^ Elk-1 could associate with inclusions present in human Huntington's Disease tissue. Huntington's Disease is characterized by intraneuronal aggregates of polyglutamine-containing Huntingtin protein. These aggregates are found within diverse brain regions in both nuclear and extranuclear compartments [24]. Ubiquitin is found within these inclusions and is commonly used as a protein marker. Figure 5A–H contains images showing that T417^+^ Elk-1 co-localizes with Ubiquitin^+^ inclusions in human Huntington's Disease tissue. Ubiquitin^+^ immunoreactivity identifies spherical inclusions within HD striatum (all arrows, Figure 5A). A subset of Ubiquitin^+^ inclusions co-localizes with T417^+^ Elk-1 (yellow arrows, Figure 5A/5B). In addition, a subset of T417^+^ Elk-1 inclusions does not contain Ubiquitin^+^ immunoreactivity (red arrows, Figure 5B). Higher magnification images demonstrate the variance in size of the inclusions (Figure 5C–H). Overall, a fraction of Ubiquitin^+^ inclusions contains T417^+^ Elk-1 and vice versa. Multiple examples of colocalization could be seen across the striatum from the HD cases listed in Table S1. Here, the degree of co-localization can vary depending on both detection and disease parameters [25].

## Discussion

The transcription factor Elk-1 resides in both nuclear and extranuclear neuronal compartments. Previously, we have shown that extranuclear Elk-1 functions in regulating neuronal viability. In this study, we identify the phosphosite T417 as being essential for Elk-1 mediated neuronal death. Furthermore, we document the co-localization of extranuclear T417^+^ Elk-1 with multiple types of neuronal inclusions from three major neurodegenerative diseases. These findings raise the interesting possibility that Elk-1 impacts neuronal viability within the context of neurodegeneration.

The finding that Elk-1 mediated neuronal death is dependent on a specific phosphosite suggests that posttranslational modifications can confer novel functional properties upon Elk-1 protein. In our *in vitro* model, the *de novo* synthesis of Elk-1 within a single dendrite leads to a local enrichment of Elk-1 protein within this subcellular compartment. This local enrichment may drive its association with a T417^+^ Elk-1 kinase that is also present in this extranuclear compartment. Similarly, the neurodegenerative microenvironment may lead to the local enrichment of extranuclear Elk-1 especially around the areas of inclusion formation. Here, this local enrichment could occur through the *de novo* synthesis of Elk-1 protein or through its subcellular movement. In addition, the neurodegenerative microenvironment may locally stimulate T417^+^ Elk-1 kinase activity.

The co-localization of T417^+^ Elk-1 with multiple neuronal inclusions suggests a common mechanism of pathogenesis and neuronal loss among distinct neurodegenerative diseases. The inclusions may sequester T417^+^ Elk-1 as it is being enriched, preventing its transport to other subcellular regions and its association with other components in the cell death pathway. However, the enrichment of this neurotoxic protein may exceed the subcellular capacity of containment resulting in the liberation of active molecules of T417^+^ Elk-1. Alternatively, the inclusions could serve as subcellular pathogenic sites that actually enhance the local enrichment and activation of T417^+^ Elk-1. T417^+^ Elk-1 molecules could then dissociate from these sites and initiate neuronal death (Figure S4). The fact that a subset of inclusions from Parkinson's Disease and Huntington's Disease show colocalization with T417^+^ Elk-1 likely reflect both disease and detection processes. Not all the neurons containing inclusion pathology likely undergo cell death or show Elk-1 enrichment at a given time. Alternatively, because limited and localized levels of Elk-1 enrichment can induce changes in cell viability [8], its possible that extranuclear Elk-1 may be biologically active even where signal can not be readily detected.

The linkage between Elk-1 and neurodegeneration raises an interesting set of issues regarding its potential as a therapeutic target[26], [27]. Its association with several types of inclusions suggests its involvement during an important stage of disease pathogenesis shared across a spectrum of neurodegenerative disease. Its ability to initiate regionalized neuronal death could be triggered by neurodegenerative processes believed to start within synaptic compartments. Also, that a specific phosphoform of Elk-1 both associates with inclusions and regulates neuronal viability suggests a molecular link from pathogenesis to cell loss dependent upon an activated form of Elk-1. Furthermore, the staining of T417^+^ Elk-1 confirms its enrichment in extranuclear compartments where it functions to regulate neuronal viability. Finally, in Alzheimer's Disease, where the staining is more intense then the canonical AT8 staining, T417^+^ Elk-1 may serve as an early biomarker for neurodegenerative processes. Currently, there are no known Elk-1 single-nucleotide poIymorphisms associated with neurodegenerative disease. However, it's likely that the role of Elk-1 in regulating neuronal viability is part of a common cellular response to regional cellular insults. In this case, SNPs may not be expected. Alternatively, genetic variants of multiple proteins involved in the Elk-1 cell death pathway could share an association with neurodegenerative disease. Accessing the absence of Elk-1 on the progression of neurodegeneration will be critical in establishing its importance in these diseases. In addition, efforts to identify components of the cell death pathway, including T417^+^ Elk-1 kinase(s) will be essential in translating these studies into novel treatment strategies.

## Materials and Methods

### Neuronal Cell Culture

Primary neurons from E18 rat hippocampi were plated in MEM (Gibco 11095-080) supplemented with 0.6% wt/vol glucose, 1 mM sodium pyruvate, and B27 (Invitrogen). The cells were placed on spiegelglas coverslips (Carolina Biological) coated with 80 µg/ml Poly-D-Lysine MW 70–150,000 (Sigma) in borate buffer and 1 µg/ml Laminin (BD Bioscience) in borate buffer.

### Site-Directed Mutagenesis

Single amino acid substitutions were placed at sites of post-translational modification using the QuikChange Site-Directed Mutagenesis kit (Stratagene). A pcDNA3.1A plasmid vector (Invitrogen) containing Elk-1 cDNA (derived from IMAGE # BC054474) was amplified using a high-fidelity DNA polymerase and PCR primers with specific single codon mutations. For the S383A mutant, the following primers were utilized: ccatttctggagcactctggcgccaattgcaccccgtagt and actacggggtgcaattggcgccagagtgctccagaaatgg. For the T417A mutant, the following primers were utilized: atcagtgtggatggcctctcggcgcccgtggtgctctccc and gggagagcaccacgggcgccgagaggccatccacactgat. Following PCR, DNA sequencing was used to verify that site-specific mutations had been created.

### Phototransfection

The capped Elk-1 mRNAs were synthesized (Ambion mMessage mMachine) and verified via a Bioanalyzer (Agilient). Prior to phototransfection, the concentration of mRNA placed in the cellular bath was 40–80 ug/ml. Phototransfection was performed on a multi-photon upright microscope (Zeiss 510 NLO) with a Ti:Sapphire laser (Mai-Tai DeepSee, Spectra Physics). Based on the transmitted light gradient contrast image or weak cellular autofluorescence of 2–3 wk IV primary hippocampal neurons, we selected three distinct regions of interest (2×2 pixel each) over a dendrite for phototransfection. Immediately following bath application of mRNA, we then used the Ti:Sapphire laser to make transient poration sites by delivering pre-chirped pulses (100 fs pulses, 80 MHz repetition rate) at 35 mW power (at the back aperture of the lens) for 25 µsec per pixel. The pulses were delivered sequentially over the pixels within the regions of interest. After phototransfection, cell viability was assessed up to 6 hours later by determining the presence of clear cellular morphological changes such as somatic swelling, dendritic fragmentation and nuclear compaction.

### Patient Information and Tissue Inclusion

Tissue used in this study was made possible by postmortem organ donation of participating patients to the University of Rochester Medical Center Neuropathology Unit. The program is rigorously administered by the Autopsy Unit of the University of Rochester Medical Center Pathology Department. HIPPA policy is in effect for the patients and their families and, as such, the cases reported here have been coded to prevent patient identification. The University of Rochester Neuropathology Unit obtained permission from the Institutional Review Board at the University of Rochester Medical Center for this study.

Following an extensive screening procedure, cases were chosen retrospectively based on their final postmortem diagnoses. Cases designated “Lewy body disease” or “Alzheimer's disease” were considered only if they showed disease-specific pathology. Cases with severe pathology were excluded due to the deficiency of neurons to assess co-localization. Age-matched controls lacking pathological changes in substantia nigra, hippocampus, and basal ganglia were also included for comparison (see Table S1 for case descriptions).

### Tissue Processing

At the time of autopsy, brain regions were carefully dissected and a 3 mm slice from the brain region of interest was placed in a tissue cassette. Cassettes were placed in formalin fixative for three days, and subsequently infiltrated with paraffin. Serial sections were cut and mounted in a similar orientation on serial Frosted Plus slides to enable identification of inclusion-containing cells and to visualize immunohistochemical results.

The data reported here are from 7 um serial section series undertaken for each of the diseases listed. All serial sets began with the first slide processed for hematoxylin and eosin using standard University of Rochester Neuropathology processing. Additional slides within the series also were processed for H&E to allow for neurons to be matched across sections. Specific neurons were located within and across adjacent sections using blood vessel morphology and tissue architecture.

### Immunohistochemistry: Parkinson's and Alzheimer's Sections

Paraffin sections were placed in fresh xylene, passed through a graded percentage of alcohol and subsequently placed in 0.15 M phosphate buffer, pH 7.4. Sections were then placed in slide mailers and processed in 3% hydrogen peroxide followed by a phosphate buffer wash. Antigen retrieval was necessary to observe immunohistochemical staining for Elk-1. Antigen retrieval was accomplished by placing the sections in 0.01 M citrate buffer at 100°C for 20 minutes in a microwave oven. Slides were cooled for 20 minutes, washed and placed in 0.15 M phosphate buffer, pH 7.4. Blocking was accomplished using a high block buffer containing 10% BSA, 10% goat serum, 0.1% Triton X-100 in 0.15 M phosphate buffer. 8 ml avidin (Vector labs Avidin/Biotin Blocking System) was added to a total of 50 ml of block buffer. Slides were blocked overnight at 4°C and then placed in primary antibody. Primary antibody dilutions are listed below. Multiple primary antibody buffers were assessed for low background staining. The final buffer used for all primary antibodies contained 5% BSA, 5% serum (from the species of the secondary antibody) and 0.1% Triton X-100 in 0.15 M phosphate buffer (“5/5” block buffer). 8 ml biotin (Vector Labs) was added to every 50 ml of “5/5” block buffer. Slides were incubated in slide mailers containing the appropriate primary antibody for 48 hours at 4°C with constant rotation. Slides containing age-matched control tissue were placed in the same slide mailers as the diseased state tissue. For each disease, slides were processed for each antibody simultaneously such that only the primary and secondary reagents were different - all other reagents and procedures were identical.

The following day, slides were removed from the primary antibody solution and washed in “5/5” block buffer. Subsequently, they were incubated in the appropriate secondary antibody in “5/5” block buffer with constant rotation for one hour at room temperature and then washed in 0.15 M phosphate buffer, pH 7.4. Avidin incubation was accomplished using the avidin/biotinylated horseradish peroxidase system (ABC Elite System) from Vector Labs. ABC was applied for one hour at room temperature with constant rotation. Slides were then washed in phosphate buffer followed by three Tris-base buffer washes. The chromagens used for detection were either diaminobenzidine (DAB) with nickel enhancement (Vector Labs DAB System) or 3-amino-9-ethylcarbazole (AEC). Intensity of the reaction product was observed using a wet microscope and stopped at the appropriate time in water. Slides containing DAB reaction product were processed through a graded series of alcohols and mounted using Cytoseal low viscosity mounting media (VWR). Slides containing AEC were mounted in the aqueous mounting media, Mowiol.

The Elk-1 primary antibodies utilized include Elk-1 (1∶500, Cell Signaling, #9182, rabbit polyclonal), S383^+^Elk-1 (1∶250, Cell Signaling, #9186, mouse monoclonal), T417^+^Elk-1 (1∶500, custom made by JE lab, rabbit polyclonal) and T368^+^ Elk-1 (1∶1000, custom made by JE lab, rabbit polyclonal). The inclusion-specific antibodies utilized include AT8 (1∶1000, Pierce, 24 hour incubation at 4C, mouse) for neurofibrillary tangles and Alpha-synuclein (1∶7500, Zymed, mouse) for Lewy bodies. The secondary antibodies utilized include biotinylated goat anti-rabbit (1∶1000, Vector Labs) and biotinylated goat anti-mouse (1∶1000, Vector Labs). Peptide competition experiments were performed for each antibody (Figure S5, Figure S6).

### Immunohistochemistry: Huntington's Sections

Paraffin sections were placed in fresh xylene, passed through a graded percentage of alcohol and subsequently placed in 0.15 M phosphate buffer, pH 7.4. Following a series of TBS washes, antigen retrieval was performed by placing the slides in 1% SDS in TBS at 37°C for 20 minutes. The slides were then extensively rinsed in a large volume of TBS at RT. Blocking was accomplished using a buffer containing 3% BSA, 10% goat serum, 2% Triton-X100 in TBS at 37°C for 75 minutes. Slides were then incubated with the appropriate primary antibody (T417^+^ Elk-1 (1∶50, custom made by JE lab, rabbit polyclonal), anti-Ubiquitin (1∶100, Chemicon, mouse monoclonal)) in a buffer containing 3% BSA in 1% TBST at 37°C for 2 hrs, then at 4°C overnight, and finally 37°C for 2 hrs. After rinses in 0.1% TBST and rinses in borate buffer, the slides were re-blocked in 2% BSA in borate buffer at RT for 15 minutes. Slides were incubated with Qdot secondary antibodies (Qdot 525 goat anti-rabbit IgG (50 nM, Invitrogen), Qdot 655 goat anti-mouse IgG (20 nM, Invitrogen)) at RT for 3 hrs and then 37°C for 1 hr. Following a series of washes in borate buffer, the slides were mounted in borate buffer for immediate imaging. Slides containing age-matched control tissue were placed in the same slide mailers as the diseased state tissue.

### Imaging

Following immunohistochemistry, sections from Parkinson's and Alzheimer's disease were analyzed using a MicroFire Camera driven by PictureFrame software on an Olympus Vanox AH-2 microscope through the University of Rochester Medical Center Confocal and Conventional Microscopy Core. Original raw data images are archived. Following Microscopy Society of America recommendations, processing of images consisted of only changes in contrast and brightness of the entire original image through PictureFrame software. Range of image processing for contrast and brightness to the entire image were between −20 and +25. Gamma was changed only for the entire image of Figure 1c to a value of 1.12. Images in Figure 2a–2c were cropped to aid in comparison across adjacent sections.

Following immunohistochemistry, sections from Huntington's disease were imaged for Qdot fluorescence using a Zeiss 510 Meta confocal microscope. Excitation at 458 nm was used to elicit Qdot emission. Qdot emission from both the Qdot 525 goat anti-rabbit IgG and the Qdot 655 goat anti-mouse IgG could be segregated across two channels with distinct bandwidths (Chs1: 511.60–533 nm; Chs2: 640–660 nm). To minimize tissue autofluorescence, a photobleaching protocol was implemented. Here, using a defined set of parameters, a region-of-interest was imaged with high-transmission, multi-wavelength excitation. This resulted in a significant reduction in tissue autofluorescence across both channels without a reduction in Qdot emission intensity.

## Supporting Information

Methods S1 (0.03 MB DOC)

Table S1 1 Includes a spectrum of disease from preclinical presentation of Lewy bodies, Parkinson's variant of Lewy body disease, Lewy body disease with neocortical involvement. 2 Standard neuropathology procedures used for severity grading. 3 Vonsattel grading system. 4 Age-matched controls were deemed free of major disease in substantia nigra, hippocampus, entorhinal cortex, and basal ganglia. * Age of onset was determined by year symptoms were recognized as being abnormal and brought to the attention of a clinician, preclinical indicates that obvious disease symptoms were not present or reported. ^ Post-mortem interval.
(0.08 MB DOC)

Table S2 Disease severity grade for Lewy Body Disease cases. Rating code: +/−  =  little neuronal loss and presence of only a few pale and Lewy bodies. +1 =  focal neuronal loss and gliosis. +2 =  multiple areas of neuronal loss and gliosis. +3 =  severe neuronal loss (cases with this severity were not included in this study due to the lack of inclusions present). * number of neurons containing Pale bodies or Lewy bodies was assessed by examining a single, diagnostic H and E stained substantia nigra section from each case.
(0.03 MB DOC)

Figure S1 S383+ Elk-1 and T368+ Elk-1 do not co-localize with Lewy body inclusions in human PD tissue. Sections containing substantia nigra were processed for S383+ Elk-1 (A) and T368+ Elk-1 (B). Hashed circles identify Lewy body inclusions. (scale bars, 20 µm; original magnification, 200× (A/B), 400× (C/D). Minimal S383+ Elk-1or T368+ Elk-1 immunoreactivity is observed within the inclusions.
(2.28 MB TIF)

Figure S2 T417+ Elk-1 localizes to both nuclear and extranuclear compartments within diverse regions of control human brain tissue. Sections from patient CON2 containing substantia nigra (A, 100x), hippocampus (C, 40x) and entorhinal cortex (E, 40x) were processed for T417+ Elk-1 (scale bars, 200 µm). Higher magnifications of insets are shown for these respective regions (B, 20 µm/400×; D, 20 µm/200×; F, 200 µm/100×). Thin arrows highlight neurons with nuclear T417+ Elk-1 immunoreactivity, thick arrows highlight neurons with extranuclear T417+ Elk-1 immunoreactivity.
(8.21 MB TIF)

Figure S3 S383+ Elk-1 and T368+ Elk-1 do not co-localize with plaque and tangle inclusions in human AD tissue. Serially adjacent sections containing CA1 hippocampus were processed for AT8 (A), S383+ Elk-1 (B) and T368+ Elk-1 (C) (scale bars, 200 µm; original magnification, 40×). The area between the two blood vessels (BV) identifies numerous AT8 immunoreactive cells. Minimal S383+ Elk-1or T368+ Elk-1 immunoreactivity is observed within the neurons in this area.
(3.74 MB TIF)

Figure S4 Proposed mechanisms linking T417+ Elk-1 inclusions with neuronal loss. Protein synthesis of Elk-1 and its phosphorylation at T417 within extracellular compartments creates a toxic protein capable of influencing neuronal viability (A) (Elk-1, orange circles; ribosomes, green circles; T417 Elk-1 kinase, blue ovals). Neurodegenerative inclusions may represent sites capable of containing toxic proteins such as T417+ Elk-1 (B1) (inclusion, large yellow circle). Alternatively, the inclusions may serve as sites allowing for further enrichment or activation of T417+ Elk-1 (B2). When enrichment mechanisms supercede inclusion containment mechanisms (C1) neuronal death is initiated (D) (mitochondria, ovals labeled “mito”). Alternatively, T417+ Elk-1 molecules break away from their sites of activation (C2) initiating neuronal death (D).
(0.44 MB TIF)

Figure S5 Signal from Elk-1 antibody is blocked by a specific peptide. Serially adjacent sections containing AD hippocampus were processed with Elk-1 primary (A), without primary antibody (B), and an Elk-1 primary: Elk-1 peptide mixture (C) (scale bars, 200 µm; original magnification, 40×).
(3.18 MB TIF)

Figure S6 Signal from pElk-1 antibodies are blocked by specific phospho-peptides. Western blot analysis identifying T417+ Elk-1 and T368+ Elk-1 as 62kD bands in C57BL/6 lysate. The lysate was probed with a pElk-1 antibody (2nd lanes), pElk-1 antibody: peptide (pep) mixture (4th lanes) and a pElk-1 antibody: phosphopeptide (P04pep) mixture (6th lanes). A 50kD loading control was placed in the alternate lanes.
(2.24 MB TIF)
