# Supplementary material for: A Neurotoxic Phosphoform of Elk-1 Associates with Inclusions from Multiple Neurodegenerative Diseases
Source: PLoS One. 2010 Feb 2;5(2):e9002. doi: 10.1371/journal.pone.0009002 (PMC2814869; doi:10.1371/journal.pone.0009002)
Supplement: Methods S1 — (0.03 MB DOC) [file pone.0009002.s001.doc]

### **Materials and Methods: Supplemental**

### Table S2: Disease severity grade for Lewy body cases

Data shown in Table 2 were obtained through counts of the number of pale bodies and the number of Lewy bodies within a diagnostic H and E stained section of substantia nigra from each Lewy body disease patient included in this study. The rating scale follows recommendations in Greenfield’s Neuropathology for severity grading. For the purposes of this study, +/- severity rating represents minimal to no appreciable neuronal loss, +1 severity rating represents focal neuronal loss and gliosis, and +2 severity rating represents multiple areas of neuronal loss and gliosis. Tissue from cases having a severity rating of +3 was not used in this study due to the paucity of Lewy bodies in such cases.

### Custom Antibody Synthesis

Elk-1 phospho-specific antibodies were produced by Covance based upon human Elk-1 protein sequence (NCBI accession # CAG47048). Phosphorylated peptides centered on T417 (CVDGLS**T**PVVLSP) or T368 (CLTPVLL**T**PSSLP) were synthesized, purified by HPLC and verified via mass spectrometry. Following KLH conjugation, the phosphorylated peptides were separately injected into rabbits and the anti-sera collected via protein A purification. The rabbit IgG was affinity purified by Pacific Immunology using both non-phosphorylated and phosphorylated columns. Concentrations of each phospho-specific antibody were quantified via ELISA.

Antibody Specificity Experiments

The specificity of Elk-1 primary antibodies was determined by peptide competition experiments (Figure S7). For immunohistochemical analysis of competition, Elk-1 primary antibody (Cell Signaling) was brought to a 1:500 dilution in primary antibody buffer. Elk-1 peptide was added at a 2:1 ratio with antibody and the mixture was incubated at RT for 24 hours. Slides containing brain tissue from human Alzheimer’s disease was placed in slide mailers and incubated with the mixture and compared with a no peptide control as well as a no primary control. Western blot analysis of competition was also performed for the T417^+^ Elk-1 primary and the T368^+^ Elk-1 primary (Figure S8).
