# Supplementary material for: A Neurotoxic Phosphoform of Elk-1 Associates with Inclusions from Multiple Neurodegenerative Diseases
Source: PLoS One. 2010 Feb 2;5(2):e9002. doi: 10.1371/journal.pone.0009002 (PMC2814869; doi:10.1371/journal.pone.0009002)
Supplement: Table S1 — 1 Includes a spectrum of disease from preclinical presentation of Lewy bodies, Parkinson's variant of Lewy body disease, Lewy body disease with neocortical involvement. 2 Standard neuropathology procedures used for severity grading. 3 Vonsattel grading system. 4 Age-matched controls were deemed free of major disease in substantia nigra, hippocampus, entorhinal cortex, and basal ganglia. * Age of onset was determined by year symptoms were recognized as being abnormal and brought to the attention of a clinician, preclinical indicates that obvious disease symptoms were not present or reported. ^ Post-mortem interval. (0.08 MB DOC) [file pone.0009002.s002.doc]

**Table S1. Disease characteristics of patient cases and controls**

| **HIPPA Case Number** | **Disease variant** | **^2^Disease**  **Grade** | **Gender** | ***Age of Onset** | **Age at death** | **PMI^** |
| --- | --- | --- | --- | --- | --- | --- |
| **^1^Lewy Body Disease** |  |  |  |  |  |  |
| *Subcortical predominant* |  |  |  |  |  |  |
| LB1 | preclinical | +/- | M | n/a | 76 | 22 |
| LB4 | preclinical | +/- | F | n/a | 79 | 12 |
| LB5 | preclinical | +1 | F | n/a | 61 | 7.5 |
| LB2 | Classical  Parkinson’s | +2 | F | --- | 83 | 8.5 |
| LB3 | Classical Parkinson’s | +1 | M | 57 | 80 | 24 |
| *Neocortical involvement* |  |  |  |  |  |  |
| LB6 | LBD + neo | +1 | M | 64 | 74 | 17 |
| LB7 | LBD + neo | +2 | M | --- | 85 | 50 |
| LB8 | LBD + neo | +2.5 | M | --- | 66 | 4 |
|  |  |  |  |  |  |  |
| **Alzheimer’s Disease** |  |  |  |  |  |  |
| AD1 | n/a | VI | M | 47 | 62 | 3.5 |
| AD2 | n/a | V-VI | F | 77 | 86 | 10 |
| AD3 | n/a | V-VI | M | 66 | 75 | 3 |
| AD4 | n/a | V-VI | M | 61 | 76 | 4 |
| AD5 | n/a | V-VI | F | 68 | 80 | 12 |
|  |  |  |  |  |  |  |
| **Huntington’s Disease** |  |  |  |  |  |  |
| HD7 | n/a | II-III**^3^** | F | 37 | 47 | n/a |
| HD8 | n/a | II**^3^** | F | 50 | 68 | 6 |
| HD9 | n/a | II-III**^3^** | F | 57 | 70 | 6 |
|  |  |  |  |  |  |  |
| **^4^Age-Matched Controls** |  |  |  |  |  |  |
| CON1 | n/a | --- | F | --- | 86 | 24 |
| CON2 | n/a | --- | F | --- | 84 | 3 |
| CON3 | n/a | --- | M | --- | 57 | 4.5 |
| CON4 | n/a | --- | M | --- | 73 | 7 |
