# Supplementary material for: A Neurotoxic Phosphoform of Elk-1 Associates with Inclusions from Multiple Neurodegenerative Diseases
Source: PLoS One. 2010 Feb 2;5(2):e9002. doi: 10.1371/journal.pone.0009002 (PMC2814869; doi:10.1371/journal.pone.0009002)
Supplement: Table S2 — Disease severity grade for Lewy Body Disease cases. Rating code: +/− = little neuronal loss and presence of only a few pale and Lewy bodies. +1 = focal neuronal loss and gliosis. +2 = multiple areas of neuronal loss and gliosis. +3 = severe neuronal loss (cases with this severity were not included in this study due to the lack of inclusions present). * number of neurons containing Pale bodies or Lewy bodies was assessed by examining a single, diagnostic H and E stained substantia nigra section from each case. (0.03 MB DOC) [file pone.0009002.s003.doc]

| **HIPPA Case Number** | ***Number of neurons**  **containing**  **Pale bodies** | ***Number of**  **neurons**  **containing**  **Lewy bodies** | **Disease Severity grade** |
| --- | --- | --- | --- |
| **Low Lewy Body Cases** |  |  |  |
| LB1 | 1 | 0 | +/- |
| LB4 | 6 | 4 | +/- |
| LB5 | 2 | 1 | +1 |
| LB2 | 10 | 6 | +2 |
| LB3 | 4 | 1 | +1 |
| **High Lewy Body Cases** |  |  |  |
| LB6 | 14 | 19 | +1 |
| LB7 | 4 | 7 | +2 |
| LB8 | 16 | 20 | +2.5 |
