# Supplementary figures and images for: A Neurotoxic Phosphoform of Elk-1 Associates with Inclusions from Multiple Neurodegenerative Diseases
Source: PLoS One. 2010 Feb 2;5(2):e9002. doi: 10.1371/journal.pone.0009002 (PMC2814869; doi:10.1371/journal.pone.0009002)

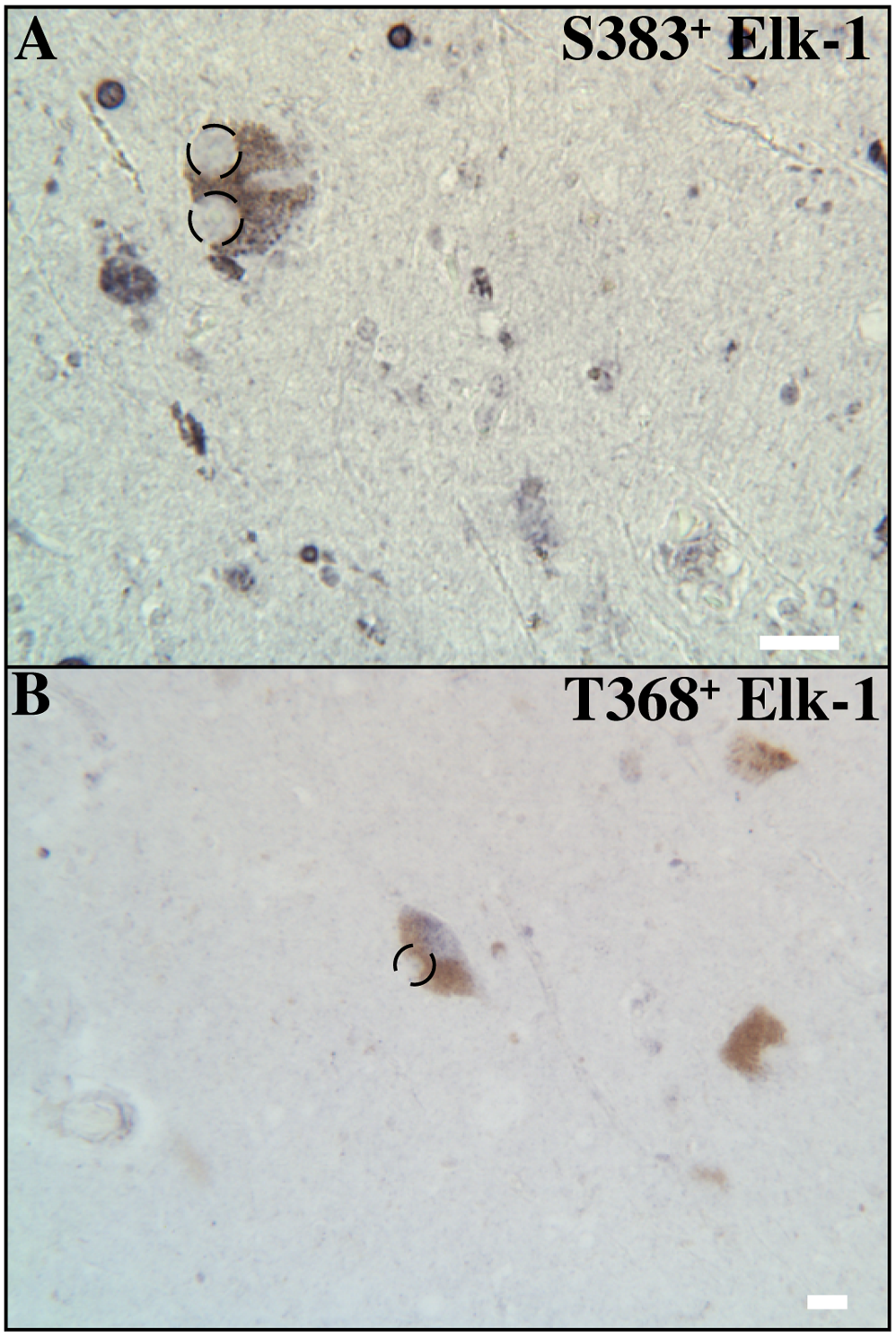

Supplement: Figure S1 — S383+ Elk-1 and T368+ Elk-1 do not co-localize with Lewy body inclusions in human PD tissue. Sections containing substantia nigra were processed for S383+ Elk-1 (A) and T368+ Elk-1 (B). Hashed circles identify Lewy body inclusions. (scale bars, 20 µm; original magnification, 200× (A/B), 400× (C/D). Minimal S383+ Elk-1or T368+ Elk-1 immunoreactivity is observed within the inclusions. (2.28 MB TIF) [file pone.0009002.s004.tif]

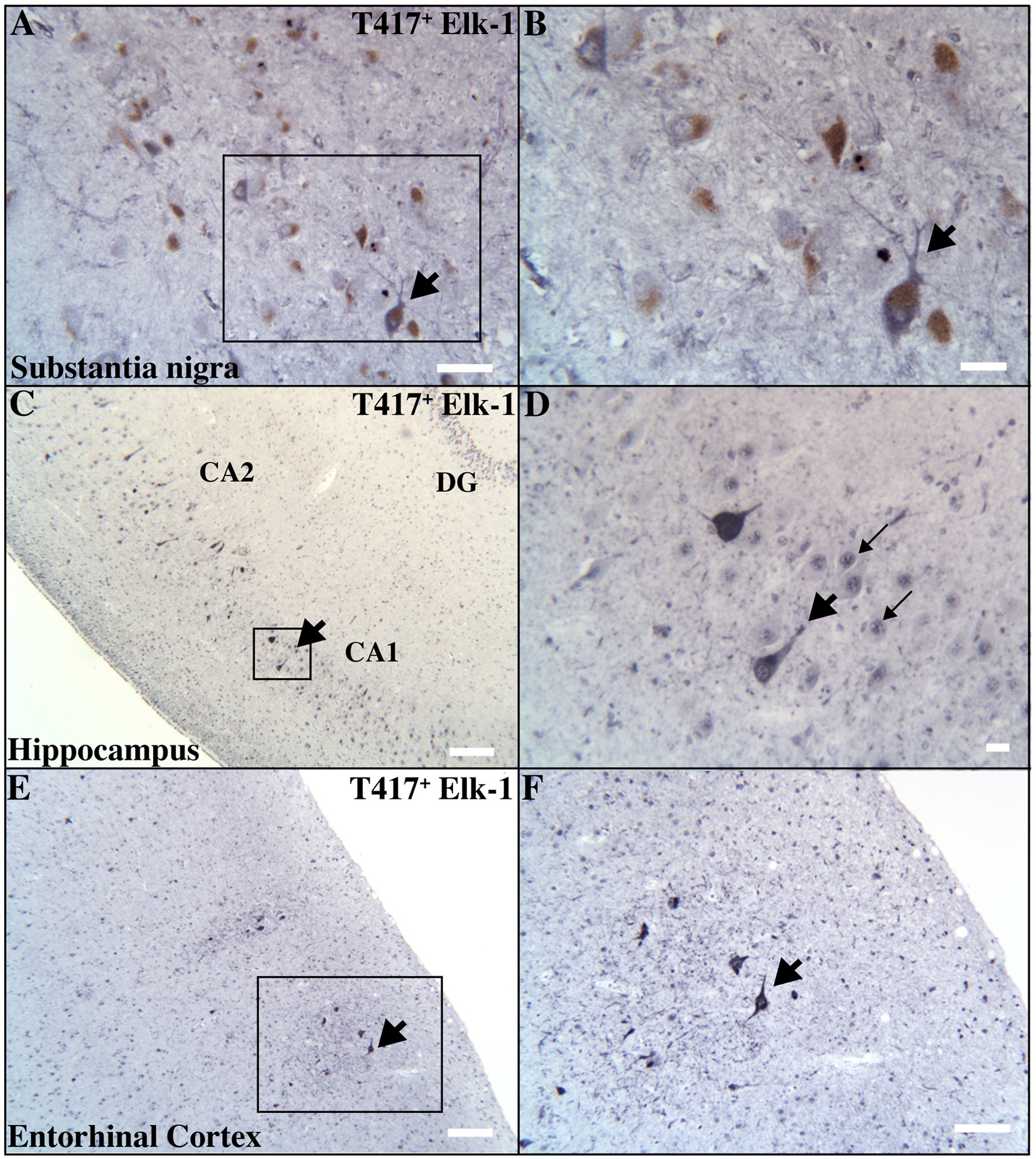

Supplement: Figure S2 — T417+ Elk-1 localizes to both nuclear and extranuclear compartments within diverse regions of control human brain tissue. Sections from patient CON2 containing substantia nigra (A, 100x), hippocampus (C, 40x) and entorhinal cortex (E, 40x) were processed for T417+ Elk-1 (scale bars, 200 µm). Higher magnifications of insets are shown for these respective regions (B, 20 µm/400×; D, 20 µm/200×; F, 200 µm/100×). Thin arrows highlight neurons with nuclear T417+ Elk-1 immunoreactivity, thick arrows highlight neurons with extranuclear T417+ Elk-1 immunoreactivity. (8.21 MB TIF) [file pone.0009002.s005.tif]

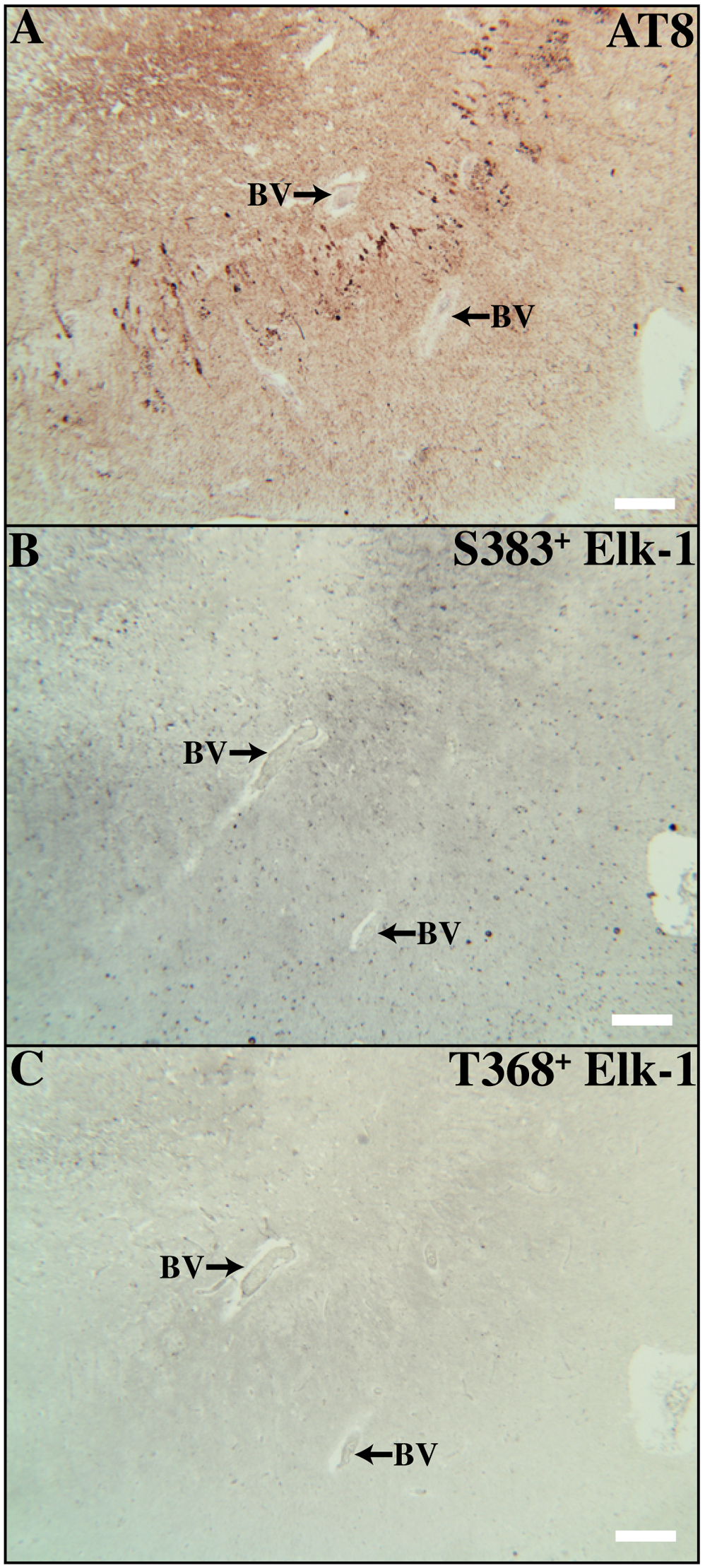

Supplement: Figure S3 — S383+ Elk-1 and T368+ Elk-1 do not co-localize with plaque and tangle inclusions in human AD tissue. Serially adjacent sections containing CA1 hippocampus were processed for AT8 (A), S383+ Elk-1 (B) and T368+ Elk-1 (C) (scale bars, 200 µm; original magnification, 40×). The area between the two blood vessels (BV) identifies numerous AT8 immunoreactive cells. Minimal S383+ Elk-1or T368+ Elk-1 immunoreactivity is observed within the neurons in this area. (3.74 MB TIF) [file pone.0009002.s006.tif]

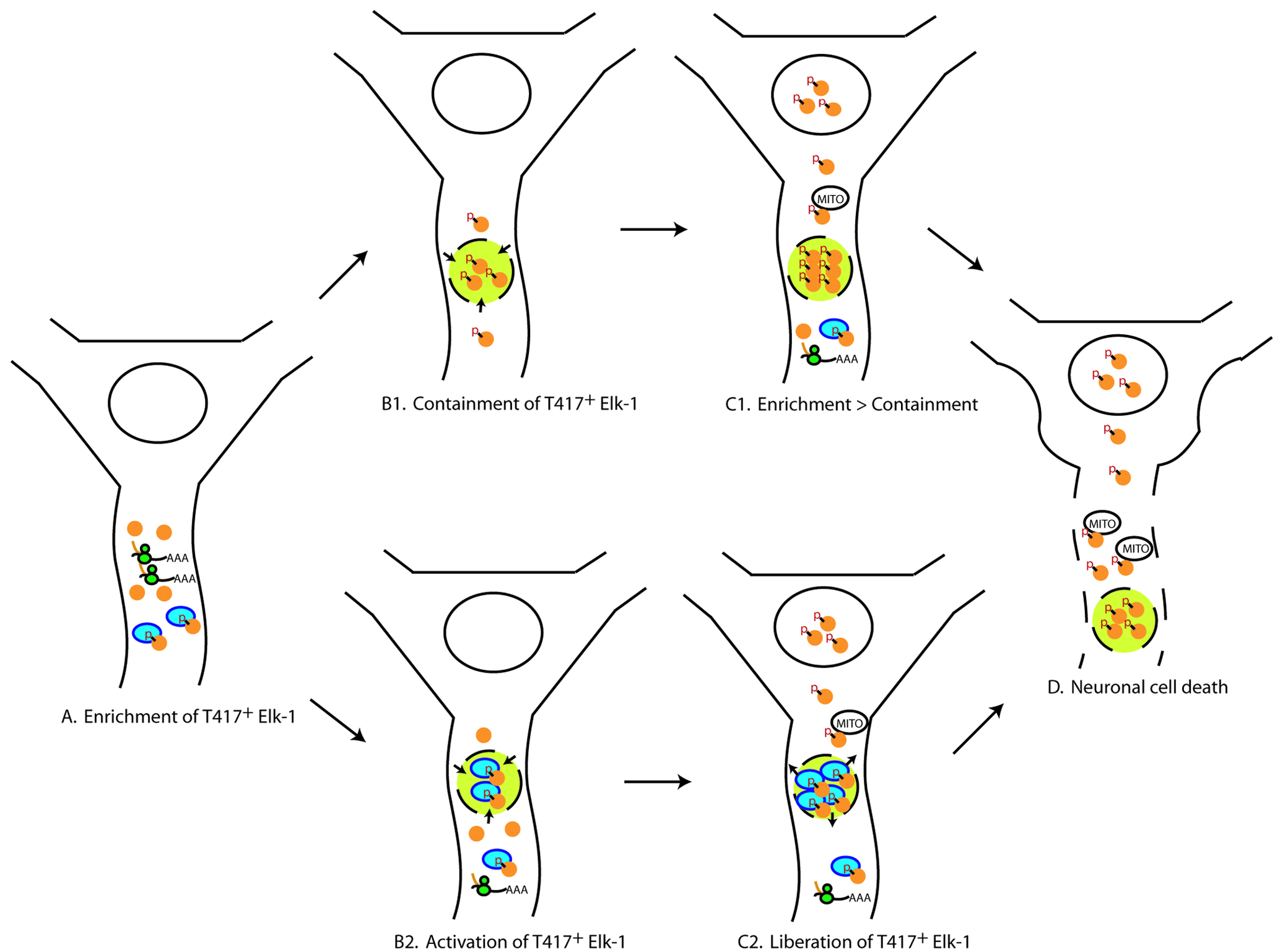

Supplement: Figure S4 — Proposed mechanisms linking T417+ Elk-1 inclusions with neuronal loss. Protein synthesis of Elk-1 and its phosphorylation at T417 within extracellular compartments creates a toxic protein capable of influencing neuronal viability (A) (Elk-1, orange circles; ribosomes, green circles; T417 Elk-1 kinase, blue ovals). Neurodegenerative inclusions may represent sites capable of containing toxic proteins such as T417+ Elk-1 (B1) (inclusion, large yellow circle). Alternatively, the inclusions may serve as sites allowing for further enrichment or activation of T417+ Elk-1 (B2). When enrichment mechanisms supercede inclusion containment mechanisms (C1) neuronal death is initiated (D) (mitochondria, ovals labeled “mito”). Alternatively, T417+ Elk-1 molecules break away from their sites of activation (C2) initiating neuronal death (D). (0.44 MB TIF) [file pone.0009002.s007.tif]

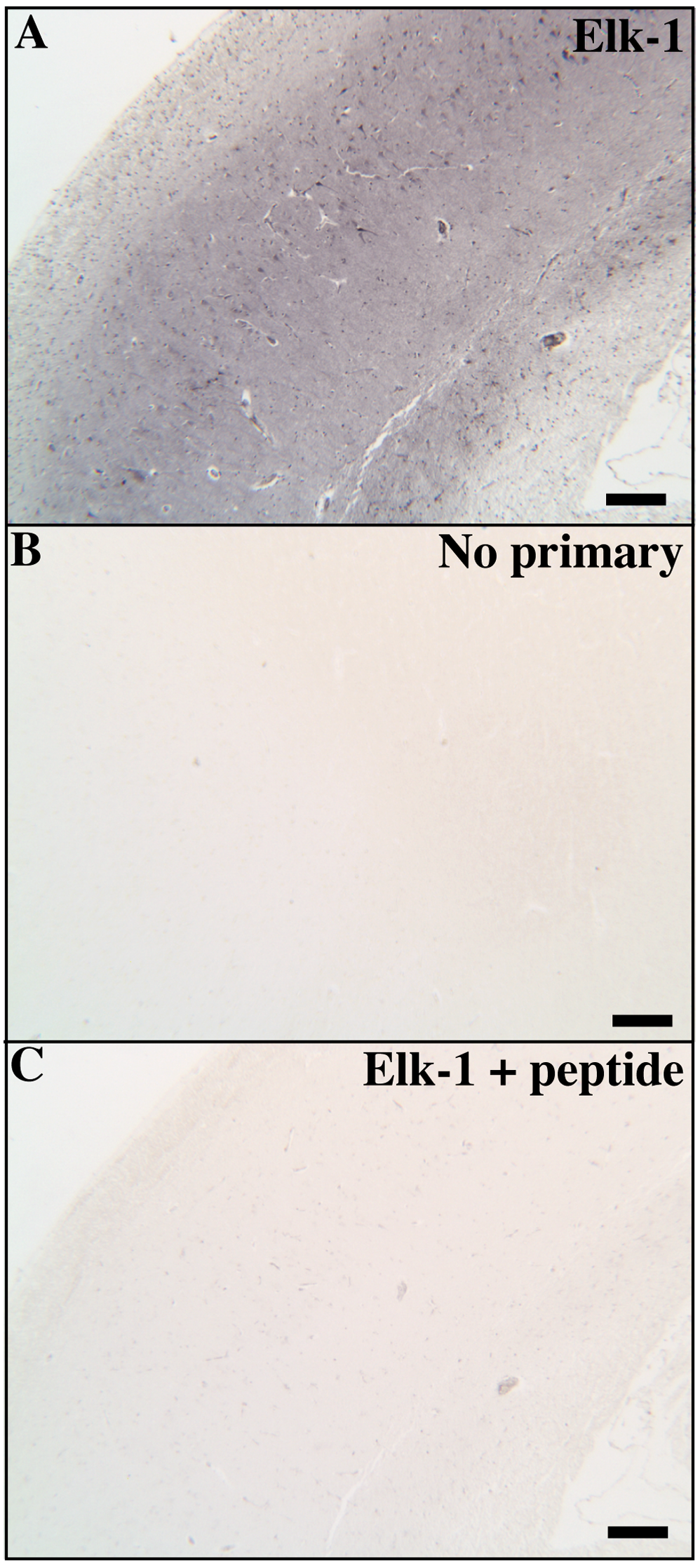

Supplement: Figure S5 — Signal from Elk-1 antibody is blocked by a specific peptide. Serially adjacent sections containing AD hippocampus were processed with Elk-1 primary (A), without primary antibody (B), and an Elk-1 primary: Elk-1 peptide mixture (C) (scale bars, 200 µm; original magnification, 40×). (3.18 MB TIF) [file pone.0009002.s008.tif]

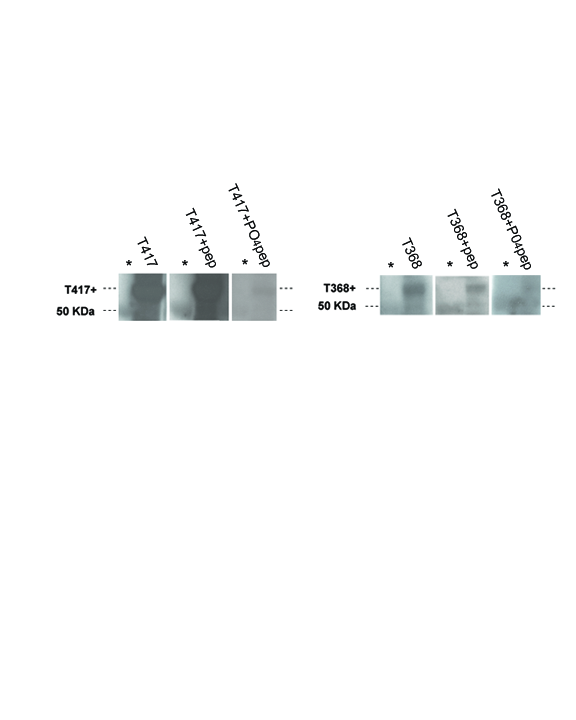

Supplement: Figure S6 — Signal from pElk-1 antibodies are blocked by specific phospho-peptides. Western blot analysis identifying T417+ Elk-1 and T368+ Elk-1 as 62kD bands in C57BL/6 lysate. The lysate was probed with a pElk-1 antibody (2nd lanes), pElk-1 antibody: peptide (pep) mixture (4th lanes) and a pElk-1 antibody: phosphopeptide (P04pep) mixture (6th lanes). A 50kD loading control was placed in the alternate lanes. (2.24 MB TIF) [file pone.0009002.s009.tif]
